# Editorial: Patterns of all-cause and cause-specific mortality during the SARS-CoV-2 pandemic: The impact of health policies and interventions

**DOI:** 10.3389/fpubh.2022.1106067

**Published:** 2022-12-06

**Authors:** Davide Golinelli, Francesco Sanmarchi, Elisa Maietti, Fabrizio Toscano, Andrea Bucci

**Affiliations:** ^1^Department of Biomedical and Neuromotor Sciences, University of Bologna, Bologna, Italy; ^2^Montefiore Medical Center, Bronx, NY, United States; ^3^Department of Economics, G. d'Annunzio University of Chieti and Pescara, Pescara, Italy

**Keywords:** mortality, COVID-19, SARS-CoV-2, pandemic, epidemiology

For many years, mortality data have been used - and still are - to describe and track the characteristics of those dying in countries worldwide and to allow comparisons of death trends between different geographical areas (1, 2). Mortality data are also required as inputs into population projections, which are used for a variety of policy-related and planning purposes. Mortality statistics provide an important indicator of the health and wellbeing of a population, as they are proxy measures of population health, for example, life expectancy, as well as to understand differentials in population health among different population subgroups. Mortality statistics also provide information about the nature and efficacy of healthcare delivery systems, which is crucial for healthcare policy-making (1–4).

During the ongoing pandemic, a surge in overall deaths has been recorded in many countries, mostly attributable to COVID-19 (5). To date, the SARS-CoV-2 outbreak is considered one of the worst health crises of the century, with over 6 million deaths globally (November 16, 2022) (6). A large number of researchers investigated the patterns of COVID-19-related excess mortality at the international, national, and subnational levels (7, 8), with most of the studies focusing on excess mortality. Apart from crude mortality, the COVID-19 pandemic had a profound impact on societies around the globe. Plenty of literature showed that the pandemic affected not only health but also the environment, economy, education, and human psychology (9). The virus outbreak determined a radical shift in people's and communities everyday life, reducing social interaction, causing employment, and determining an overall increase in symptoms like distress, depression, anxiety, and frustration (10).

The COVID-19 pandemic has also resulted in major shifts in patterns of healthcare consumption and delivery and had a substantial effect on the use of and access to essential healthcare services, exacerbating morbidity and mortality from other diseases, in addition to direct COVID-19 mortality (11). Health systems worldwide faced significant challenges in providing essential health services, with reported disruptions in over 90% of countries surveyed in the third round of WHO's Global pulse survey on the continuity of essential health services during the COVID-19 pandemic (12). Disruption of health services occurred in all major health areas including sexual, reproductive, maternal, newborn, child, and adolescent health, immunization, nutrition, cancer care, mental, neurological and substance use disorders, HIV, hepatitis, TB, malaria, neglected tropical diseases, and care for older people.

Starting from the 1^st^ months of 2020, researchers around the globe started to investigate COVID-19-related mortality data (13–16). Conceivably, the pandemic breathed new life into epidemiological research and researchers, who were able to take advantage of the opportunity provided by COVID-19 data collected (almost) globally. As the pandemic progressed, researchers began to describe the virus's impact on non-COVID-19 mortality (e.g., deaths related to the variation of the quality of care provided to the patients, and to the stress suffered by healthcare systems) (13–16).

In this Research Topic, we looked for studies analyzing publiclyavailable data, aggregated data, data generated by healthcare systems, and other data, comparing mortality patterns, investigating the spatio-temporal trends and their possible determinants, addressing the burden of the pandemic on the population, through methodologically sound study design and analyses. The importance of this Research Topic has been stressed by Pashkevich and Burghardt, who delivered an important message, warning the world's scientific community to be aware of the thousands of silent deaths of those who were very likely the victims of COVID-19 despite not being diagnosed with it. Concerning the number of lives lost during this health crisis, it is essential to mention the interesting study by Rousson and Locatelli which aimed to quantify the (direct and indirect) impacts of the COVID-19 pandemic on mortality for actual populations of persons living in 12 European countries in 2020. They calculated a “population life loss” in 2020 for men and women living in several European countries, basing their analysis on demographic and mortality data, as well as remaining life expectancies found in the Human Mortality Database. The Authors concluded that analyses based on the concept of population life loss are consistent with those based on the classical concept of life expectancy, confirming the significant impact of COVID-19 on mortality.

As reported before, burgeoning literature confirmed the excessive number of deaths, directly and indirectly caused by the virus, despite the countermeasures implemented by individuals, organizations, and national governments. On this subject, Capodici et al. analyzed to which extent the number and strictness of government policies in six countries reduced excess deaths associated with COVID-19 as well as peoples' mobility, used here as a proxy for compliance with COVID-19 restrictions. Their study suggested that the governments' non-pharmaceutical interventions set up in Western Europe and the US to cope with the pandemic were respected by most citizens and, as a result, contributed to alleviating the burden of the pandemic on health services.

The changing trends of patient characteristics during the COVID-19 pandemic have been another topic of interest. The analysis conducted by König et al. on more than seventy thousand inpatient cases compared patient characteristics and outcomes of the first wave of the pandemic with the periods thereafter up to mid-2022. It showed the trends toward a reduction of mean age and the presence of relevant comorbidities as well as in-hospital mortality in inpatients with proven SARS-CoV-2 infection.

The evidence reported in this Frontiers in Public Health Research Topic confirms the important work of the global scientific community in describing the impact of the SARS-CoV-2 pandemic in terms of patterns of all-cause and cause-specific mortality. This effort is crucial, as where information on the cause of death is recorded, data on mortality can help policymakers understand a country's trajectory through the epidemiological transition caused by a health emergency.

## Author contributions

DG, FS, FT, EM, and AB have jointly and equally contributed to drafting the manuscript and describing contributions out of which this Research Topic consisted. All authors deserve fully their authorship based on the intellectual content they provided and joint workflow during the topic development.
